# Urotensin II in the development and progression of chronic kidney disease following ⅚ nephrectomy in the rat

**DOI:** 10.1113/EP087366

**Published:** 2019-01-15

**Authors:** Heather J. Eyre, Thomas Speight, Jocelyn D. Glazier, David M. Smith, Nick Ashton

**Affiliations:** ^1^ Divison of Pharmacy and Optometry Faculty of Biology Medicine and Health University of Manchester Manchester UK; ^2^ Maternal and Fetal Health Research Centre Division of Developmental Biology and Medicine University of Manchester St Mary's Hospital Manchester UK; ^3^ Discovery Sciences Innovative Medicines & Early Development Biotech Unit AstraZeneca Cambridge Science Park Cambridge UK; ^4^ Division of Cardiovascular Sciences Faculty of Biology Medicine and Health University of Manchester Manchester UK

**Keywords:** chronic kidney disease, subtotal nephrectomy, urotensin II

## Abstract

**New Findings:**

- **What is the central question of this study?**
  Urotensin II is upregulated in patients in the later stages of chronic kidney disease (CKD), particularly in individuals requiring dialysis. Could treatment with a urotensin II receptor antagonist slow progression of renal disease?
- **What is the main finding and its importance?**
  In the rat, expression of urotensin II and its receptor increased, extending into cortical structures as CKD progressed towards end‐stage renal failure. Subchronic treatment with a urotensin receptor antagonist slowed but did not prevent progression of CKD. This suggests that urotensin II contributes to the decline in renal function in CKD.

**Abstract:**

Elevated serum and urine urotensin II (UII) concentrations have been reported in patients with end‐stage chronic kidney disease (CKD). Similar increases in UII and its receptor, UT, have been reported in animal models of CKD, but only at much earlier stages of renal dysfunction. The aim of this study was to characterize urotensin system expression as renal disease progresses to end‐stage failure in a ⅚ subtotal nephrectomy (SNx) rat model. Male Sprague–Dawley rats underwent SNx or sham surgery and were killed at 8 weeks postsurgery [early (E)] or immediately before end‐stage renal failure [30 ± 3 weeks postsurgery; late (L)]. Systolic blood pressure, urinary albumin:creatinine ratio and glomerulosclerosis index were all increased in SNx‐E rats compared with sham‐E by 8 weeks postsurgery. These changes were associated with an increase in renal immunoreactive UII staining but little change in UT expression. As CKD progressed to end‐stage disease in the SNx‐L group, markers of renal function deteriorated further, in association with a marked increase in immunoreactive UII and UT staining. Subchronic administration of a UT antagonist, SB‐611812, at 30 mg kg^−1^ day^−1^ for 13 weeks, in a separate group of SNx rats resulted in a 2 week delay in the increase in both systolic blood pressure and urinary albumin:creatinine ratio observed in vehicle‐treated SNx but did not prevent the progression of renal dysfunction. The urotensin system is upregulated as renal function deteriorates in the rat; UT antagonism can slow but not prevent disease progression, suggesting that UII plays a role in CKD.

## 1 INTRODUCTION

The mammalian urotensin system comprises two structurally homologous peptides, urotensin II (UII) and urotensin II‐related peptide (URP), and a single G_q/11_‐linked receptor, UT. Despite the similarity of the mature proteins, UII and URP are the products of two different genes, *Uts2* (Coulouarn, Jegou, Tostivint, Vaudry, & Lihrmann, 1999) and *Uts2b* (Sugo & Mori, 2008) respectively. Urotensin II was shown initially to be a potent vasoconstrictor in mammals (Ames et al., 1999), but it is now known to exert a wide range of influences, including a regulatory role in the kidney (Forty & Ashton, 2012; Song et al., 2006). Indeed, the kidney is a major source of UII in both humans (Shenouda, Douglas, Ohlstein, & Giaid, 2002) and rats (Song et al., 2006); in contrast, the primary source of URP is the brain (Sugo et al., 2003), although it is also found in the kidney (Song et al., 2006). UT receptors have been localized to the human (Matsushita et al., 2001) and rat (Abdel‐Razik, Forty, Balment, & Ashton, 2008b; Forty & Ashton, 2012; Song et al., 2006) kidney, with greater abundance in the medulla compared with the cortex (Abdel‐Razik et al., 2008b; Song et al., 2006).

Elevated concentrations of UII have been reported in patients with a variety of renal diseases, including IgA nephropathy and diabetic nephropathy (Balat, Karakok, Yilmaz, & Kibar, 2007; Garoufi et al., 2017; Langham et al., 2004; Mosenkis et al., 2011; Totsune et al., 2001, 2004; Woo et al., 2010). Serum or urinary UII concentrations are higher in patients with more severe renal dysfunction, which has led to the suggestion that UII plays a role in chronic kidney disease (CKD).

Chronic kidney disease is characterized by the development of glomerulosclerosis, which arises through enhanced activity of myofibroblasts, leading to an accumulation of abnormal extracellular matrix components (Liu, 2006). Urotensin II has been shown to promote fibrosis in the heart (Bousette et al., 2006; Chen et al., 2008; Dai et al., 2007; Tran et al., 2010; Tzanidis et al., 2003), blood vessels (Zhao et al., 2013), lungs (Onat et al., 2012) and liver (Kemp et al., 2009; Liu et al., 2010). In streptozotocin‐induced diabetic rats, UII and UT were upregulated in association with an increase in renal fibrosis and the accumulation of extracellular matrix components (Tian et al., 2008). Furthermore, rats subject to ⅚ subtotal nephrectomy exhibited increased UII and UT mRNA expression in the remnant kidney at a time when plasma creatinine concentrations were increased fivefold, indicative of impaired glomerular filtration (Mori et al., 2009).

Although components of the urotensin system have been reported to be upregulated in rodent models of renal impairment, there is a mismatch between current experimental data and patient profiles in terms of disease progression. The clinical reports of elevated UII associated with renal disease have been based on patients with marked reductions in glomerular filtration rate or those requiring dialysis (CKD stages 4 and 5). In contrast, animal studies to date have been conducted at much earlier stages in renal disease progression (CKD stages 2 and 3). Although these animal studies have been able to provide some mechanistic insight, they are not representative of the CKD patient with marked impairment of renal function.

Accordingly, the aims of this study were to characterize urotensin system expression in the rat kidney as renal disease progressed from early dysfunction to end‐stage renal failure and to determine whether antagonism of the UT receptor could slow or prevent the progression of CKD in a ⅚ subtotal nephrectomy model.

## 2 METHODS

### 2.1 Ethical approval

The animal experiments were conducted under the authority of a licence (PPL 40/3438) granted in accordance with the UK Animals (Scientific Procedures) Act 1986 and received local ethical approval.

### 2.2 Subtotal nephrectomy procedure

Male Sprague–Dawley rats (8–10 weeks, 285 ± 28 g) were purchased from Charles River (Margate, UK) and held in the animal facility for 1 week before use. Animals had access to a standard diet (RM1, expanded pellet diet; Special Diet Services, Witham, UK) and water *ad libitum* at all times, and were held in a 12 h–12 h light–dark cycle with controlled temperature (22–24°C) and relative humidity.

Subtotal nephrectomy (SNx) was carried out as a two‐step surgical procedure to reduce renal mass. This approach was chosen instead of the alternative, which involves ligating branches of the renal artery, because the latter induces a marked increase in activity of the renin–angiotensin system (Yang, Zuo, & Fogo, 2010). In the first stage, the upper and lower poles of the left kidney were removed under isoflurane anaesthesia (2% isoflurane in oxygen at 2 l min^−1^), leaving one‐third of the left kidney intact. The cut faces were sealed with tissue glue [Histoacryl (*n*‐butyl‐2‐cyanoacrylate); Braun Medical Ltd, Sheffield, UK] in order to prevent bleeding. Buprenorphine analgesia (0.06 mg per procedure; Sogeval UK, Sheriff Hutton, York, UK) and fluid replacement (1 ml, 154 mm sodium chloride for injection; Braun Medical Ltd) were administered s.c. before recovery from the anaesthetic. One week later, the right kidney was removed completely under isoflurane anaesthesia; analgesia and fluid replacement were provided as described above. A separate group of sham‐operated animals, in which the left and 1 week later the right kidney were exteriorized and returned to the peritoneal cavity, acted as controls. All animals were monitored closely over the first 48 h postsurgery for signs of acute renal failure. A total of 54 rats underwent the SNx procedure and 31 rats underwent the sham procedure. Nine rats from the SNx group were culled owing to acute renal failure (*n* = 3) or for other reasons (*n* = 6); one sham‐operated rat was culled because of over‐grooming of the surgical wound.

### 2.3 Progression to end‐stage renal failure

In order to characterize urotensin system expression as renal disease progressed, separate groups of SNx rats and time‐matched sham control animals were followed to the stage of renal dysfunction (early, 8 weeks postsurgery; SNx‐E *n* = 12 and sham‐E *n* = 6) or (near) end‐stage renal failure (late, 30 ± 3 weeks postsurgery; SNx‐L *n* = 15 and sham‐L *n* = 12). The humane endpoint for the late SNx group was determined by a combination of behavioural changes and a rapid loss of body weight (>15% compared with peak weight). Using these criteria, all SNx rats were culled before death occurred as a result of renal failure.

Systolic blood pressure (SBP) was measured every 2–4 weeks by tail‐cuff plethysmography (model LE 5002; Panlab S.L.U., Barcelona, Spain) without the use of restraint. Spot urine samples were collected every 2–4 weeks for the determination of albumin and creatinine concentrations by housing the animals in metabolism cages (Tecniplast UK Ltd, Leicester, UK) until the necessary minimum volume of urine was produced (0.5 ml, typically voided over 1–2.5 h).

At 8 weeks postsurgery for the early groups and once the defined endpoint had been reached for the late group, SNx and time‐matched sham animals, terminal blood, urine and tissue samples were collected under Inactin anaesthesia (100 mg kg^−1^ thiobutabarbital sodium; Sigma). Blood was collected by cardiac puncture into a chilled, heparinized syringe, and plasma was separated by centrifugation at 2000*g* for 10 min at 4°C and stored at −80°C before analysis; urine was collected into a chilled syringe by direct puncture of the bladder and stored at −20°C before analysis. In the SNx animals, the remnant kidney was either perfusion fixed with 4% paraformaldehyde or flash frozen in liquid nitrogen. In the sham animals, the left kidney was perfusion fixed and the right kidney was frozen.

### 2.4 The UT antagonist

**Figure 1:**
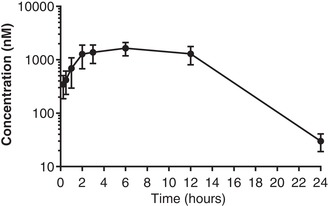
Pharmacokinetic profile of SB‐611812. Concentration of SB‐611812 in rat blood, after administration p.o. at 5 mg kg^−1^. The concentration peaked at 1643 nm 6 h after dosing. Data are shown as means ± SD from *n* = 4 rats

The UT antagonist SB‐611812 {2,6‐dichloro‐*N*‐[4‐chloro‐3‐[2‐(dimethylamino) ethyloxy] phenyl]‐4‐(trifluoromethyl) benzenesulfonamide} was chosen on the basis of a previous *in vivo* study, in which 100% bioavailability and a 4–5 h half‐life after administration by gavage were demonstrated (Rakowski et al., 2005). The bioavailability and clearance profile of SB‐611812 were also confirmed in our hands using a 24 h pharmacokinetic protocol (Figure 1). Biological efficacy was demonstrated in the rat *in vivo* after gavage administration at a dose of 30 mg kg^−1^ day^−1^ (Rakowski et al., 2005). The antagonist was synthesized by Pharmaron Beijing Co. Ltd (Beijing, China).

### 2.5 Subchronic UT antagonist treatment

Seven days after undergoing SNx or sham surgery, rats began a 13 week treatment course with either the UT antagonist SB‐611812 or vehicle [0.5% hydroxyl‐propyl methylcellulose (Sigma) with 0.1% Tween20 (Sigma) in deionized water]. Antagonist‐treated rats (SNx‐A *n* = 10, sham‐A *n* = 6) received 30 mg kg^−1^ day^−1^ of SB‐611812 by gavage (p.o.) in a vehicle volume of 2.5 ml kg^−1^; vehicle‐treated rats (SNx‐V *n* = 8, sham‐V *n* = 6) received 2.5 ml kg^−1^ of the vehicle alone (p.o.). Body weight was recorded every 2–4 days; systolic blood pressure was measured as described above every 2 weeks. Urine samples were collected as described above every 2 weeks for the measurement of albumin and creatinine. At the end of the treatment period (14 weeks postsurgery), the rats were anaesthetized with Inactin (100 mg kg^−1^) to enable the collection of terminal blood and urine samples and tissue harvesting as described above.

### 2.6 Urine and plasma analysis

Creatinine concentrations in urine and plasma samples were quantified using a colorimetric assay based upon the Jaffe reaction, according to the manufacturer's instructions (DetectX urinary creatinine kit; Arbor Assays, Ann Arbor, MI, USA).

Urinary albumin concentrations were determined by enzyme‐linked immunosorbent assay (ELISA). Flat‐bottomed 96‐well microplates were precoated with unconjugated sheep anti‐rat albumin antibody (1 mg ml^−1^ diluted 1:100; Bethyl Laboratories, Inc., Montgomery, TX, USA; catalogue no. A110‐134A, RRID:AB_185528) and blocked with 200 μl per well 1% bovine serum albumin (Melford, Ipswich, UK) in 50 mm Tris base, 140 mm sodium chloride at pH 8 in deionized water (Sigma) overnight at 4°C. Albumin standards (0–1000 ng ml^−1^ from rat reference serum, 30 mg ml^−1^ albumin; Bethyl Laboratories, Inc.) and urine samples (diluted 1:100–1:1,000,000) were incubated at room temperature (22–25°C) for 1 h. After aspiration and washing, 100 μl per well horseradish peroxide (HRP)‐conjugated sheep anti‐rat albumin detection antibody (1 mg ml^−1^ diluted 1:50,000; Bethyl Laboratories, Inc.; catalogue no. A110‐134P, RRID:AB_185529) was added for 1 h. After washing, 100 μl per well TMB substrate (1‐Step Ultra TMB reagent; Pierce, Rockford, IL, USA) was added for 15–30 min before stopping the reaction with 100 μl per well ELISA stop solution (Sigma). The optical density of the resulting yellow substrate was read at 450 nm with 570 nm correction (Biotek Synergy HT plate reader with Gen5 software).

Blood urea nitrogen was measured using an enzymatic colorimetric assay kit according to the manufacturer's instructions (Stanbio Laboratory, Boerne, TX, USA).

### 2.7 Glomerulosclerosis index

Paraffin‐embedded kidneys sectioned at 6 μm thickness were stained with Schiff's reagent [0.5% basic fuchsin (Sigma) with 1% potassium metabisulphite in 0.1 m hydrochloric acid] and counterstained with Gill III Haematoxylin (Merck Millipore, Feltham, UK) using standard procedures. Fifty glomeruli per section (*n* = 3–5 sections per experimental group) were given a glomerulosclerosis index (GSI) score of 0–4, based upon the extent of the lesions present, by an operator blinded to the groups. Sclerotic lesions were scored as 0 = no lesion was present, 1 = <25% affected, 2 = 25–50% sclerotic, 3 = 50–75% glomerular area affected, and 4 = full glomerular closure, >75% affected (el Nahas, Bassett, Cope, & Le Carpentier, 1991).

### 2.8 Immunohistochemistry

Urotensin peptides (UII and/or URP) were localized in 6‐μm‐thick sections using a polyclonal rabbit anti‐flounder UII antibody [1:100 dilution, raised against the conserved amino‐acid sequence Cys‐Phe‐Trp‐Lys‐Tyr‐Cys by Dr P. Ingleton, University of Sheffield, UK (Winter, Hubbard, McCrohan, & Balment, 1999), RRID:AB_2744516], followed by application of a secondary polyclonal goat anti‐rabbit antibody (1:100; DakoCytomation, Ely, UK; catalogue no. P0448, RRID:AB_2617138). A polyclonal goat anti‐rat UT receptor antibody (1:100 dilution; Santa Cruz Biotechnology, Santa Cruz, CA, USA; catalogue no. sc‐10194 RRID:AB_647559), followed by a polyclonal rabbit anti‐goat (1:100; DakoCytomation; catalogue no. P0449, RRID:AB_2617143) secondary antibody were used to localize UT protein in kidney sections. 3,3′‐Diaminobenzidine (ImmPACT enhanced DAB substrate; Vector Laboratories, Peterborough, UK) was used to develop antibody–HRP conjugate. Negative controls consisted of omitting primary antibody. Qualitative descriptions of the changes in location of immunoreactive proteins are provided, because DAB staining is not a quantitative method.

### 2.9 Quantitative RT‐qPCR for basement membrane components

Frozen samples of renal cortex were transitioned into RNA*later*‐ICE (Ambion, Paisley, UK) before extraction of total RNA using an RNeasy plus mini kit (Qiagen Ltd, Manchester, UK). RNA extracts were treated with gDNA wipe‐out buffer (Qiagen) before reverse transcription using QuantiScript reverse transcriptase (Qiagen). Messenger RNA for *Col4a1* (collagen IV alpha 1), *Lamb1* (laminin‐β_1_) and *Fn1* (fibronectin 1) were quantified in a 1:100 dilution of cDNA using a Stratgene Mx3000/3005P real‐time PCR machine (Agilent Technologies, Stockport, UK) and QuantiFast SYBR Green PCR mix (Qiagen) with ROX reference dye. Primers were provided in QuantiTect Primer Assay kits QT01620073, QT01574531 and QT00179333 (Qiagen) for *Col4a1*, *Lamb1* and *Fn1*, respectively. Messenger RNAs were quantified against standard curves generated from pooled cDNA and normalized to expression of the housekeeping genes *Ywhaz* (tyrosine 3‐monooxygenase/tryptophan 5‐monooxygenase activation protein, zeta) and *B2m* (β2 microglobulin).

### 2.10 Statistical analysis

Data are presented as the mean ± SD for normally distributed continuous variables or the median (interquartile range) for ordinal data or continuous data that were not normally distributed (Shapiro–Wilk test). Statistical analysis was by two‐way or three‐way ANOVA with repeated measures, with Tukey's *post hoc* tests, according to the number of independent variables. Urinary albumin:creatinine ratio (uACR) and mRNA data were subject to log_10_‐transformation before ANOVA. Glomerulosclerosis index scores were compared using a Kruskal–Wallis test, with Mann–Whitney *post hoc* tests adjusted by Holm's sequential Bonferroni correction for multiple comparisons. Analysis was performed using SPSS (version 22.0; IBM SPSS Statistics, IBM United Kingdom Ltd, Portsmouth, UK); statistical significance was ascribed at the 5% level.

## 3 RESULTS

### 3.1 Progression of CKD in the SNx rat

The SNx‐E rats started to develop increased SBP compared with the sham‐operated control animals by 7 weeks postsurgery (sham‐E 141.9 ± 4.7 mmHg, *n* = 6 *versus* SNx‐E 154.5 ± 10.0 mmHg, *n* = 12, mean ± SD, *P* = 0.02); however, this was not allowed to progress further, because terminal samples were collected at 8 weeks postsurgery. The SNx‐L rats developed systolic hypertension over the course of the study; in contrast, there was no change in SBP in the sham control animals. The SBP was significantly higher in the SNx‐L group compared with the sham control animals by 12 weeks postsurgery (sham‐L 132.0 ± 7.6 mmHg, *n* = 12 *versus* SNx‐L 156.7 ± 9.7 mmHg, *n* = 15, *P* < 0.001). This hypertension was progressive, increasing in severity with time (*P*_time_ < 0.001), reaching 175.4 ± 18.0 mmHg after 28 weeks (SNx‐L, *n* = 10) compared with 141.3 ± 8.4 mmHg (*n* = 9) in the sham‐L group (Table 1). The mean survival time in SNx rats allowed to progress to end‐stage renal failure was 30 ± 11.6 weeks.

**Table 1:** Systolic blood pressure, terminal urine and plasma analysis and glomerulosclerosis index scores in subtotal nephrectomized (SNx) and sham‐operated rats

	Early; renal dysfunction		Late; end‐stage renal failure	
Parameter	Sham‐E	SNx‐E	Sham‐L	SNx‐L
Systolic blood pressure (mmHg)	141.9 ± 4.7 [6]	154.5 ± 10.0 [12]*	141.3 ± 8.4 [9]	175.4 ± 18.0 [10]***
Urinary albumin:creatinine ratio (mg μmol^−1^)	0.04 (0.02–0.05) [3]	0.79 (0.45–1.96) [11]*	0.13 (0.05–0.28) [12]	3.55 (1.55–6.13) [13]***
Plasma creatinine (μmol l^−1^)	34.5 ± 11.0 [6]	89.6 ± 22.2 [11]	62.7 ± 20.4 [12]	351.3 ± 137.4 [13]***
Blood urea nitrogen (mmol l^−1^)	5.2 ± 1.5 [6]	18.7 ± 6.0 [11]*	9.8 ± 4.2 [12]	66.2 ± 13.3 [13]***
Glomerulosclerosis index (a.u.)	0 (0–0) [5]	1 (0–1) [3]***	0 (0–0) [5]	3 (2–4) [5]***

Systolic blood pressure was measured at 7 weeks in the early (E) group and at 28 weeks postsurgery in the late (L) group. Terminal samples were collected at 8 weeks in the early group and when the SNx rats reached the defined humane endpoint (mean 30 ± 11.6 weeks postsurgery) in the late group; a time‐matched sham animal was killed at the same point. The number of animals per group is indicated in square brackets. Data are shown as means ± SD for normally distributed continuous variables or medians (interquartile range) for ordinal data or those that were not normally distributed. Statistical comparisons were by two‐way ANOVA with Tukey's *post hoc* tests or Kruskal–Wallis test with Mann–Whitney *post hoc* tests corrected for multiple comparisons. For clarity, comparisons are shown only between time‐matched SNx and sham animals. ^*^ *P* < 0.05, ^***^ *P* < 0.001 *versus* time‐matched sham.

Urinary albumin:creatinine ratio increased in the SNx animals over the course of the experiment (*P*_time_ < 0.001), becoming significantly greater than that of the control rats by 7 weeks postsurgery [sham‐L 0.01 (0.002–0.01) mg μmol^−1^, *n* = 12 *versus* SNx‐L 0.06 (0.02–0.94) mg μmol^−1^, *n* = 15, median (interquartile range), *P* = 0.007] and remained elevated for the remainder of the study. The uACR in terminal urine samples were also significantly higher in the SNx animals by comparison with the sham control animals (Table 1).

Although terminal plasma creatinine in the SNx‐E group was not statistically different from that of the sham‐E control group (*P* = 0.52), blood urea nitrogen was significantly higher in SNx‐E animals (*P* = 0.014; Table 1). In the SNx‐L group, both plasma creatinine (*P* < 0.001) and blood urea nitrogen (*P* < 0.001) were significantly higher than in the sham‐L group.

**Figure 2:**
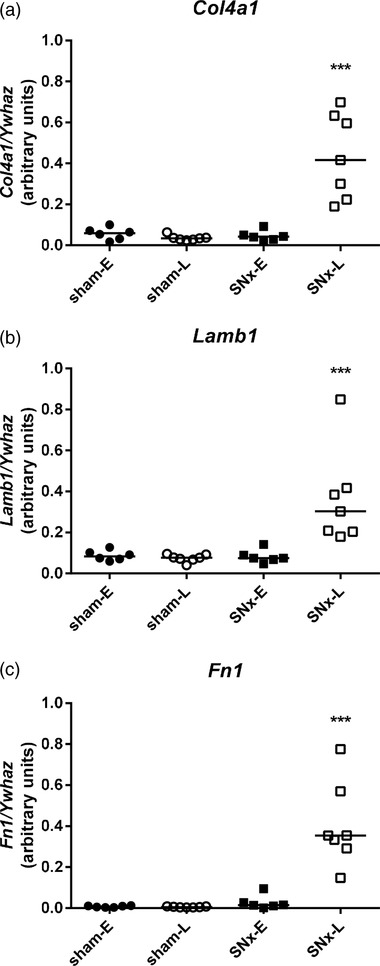
Collagen IV α1 (*Col4a1*; a), laminin‐β_1_ (*Lamb1*; b) and fibronectin 1 (*Fn1*; c) mRNA expression normalized to *Ywhaz* in the renal cortex of rats after subtotal nephrectomy (SNx), as chronic kidney disease progresses. Glomerular basement membrane marker expression is shown at 8 weeks postsurgery [early (E)] and at end‐stage renal failure [late (L)]. Data are shown as a scatter plot with median; statistical comparisons were by two‐way ANOVA of log_10_‐transformed data followed by Tukey's *post hoc* test; ^***^ *P* < 0.001 SNx‐L *versus* sham‐L

The glomerulosclerosis index was significantly higher in both SNx‐E (*P* < 0.001) and SNx‐L (*P* < 0.001) compared with their respective controls (Table 1). Glomerular basement membrane marker mRNAs *Col4a1* (*P* = 0.95), *Lamb1* (*P* = 0.99) and *Fn1* (*P* = 0.27) were not significantly different in the renal cortex of SNx‐E rats compared with sham‐E. However, expression of these markers was significantly upregulated in the SNx‐L animals compared with time‐matched shams (*P* < 0.001; Figure 2).

### 3.2 Renal UII and UT protein expression increases as CKD progresses

In common with all commercially available antibodies, the UII antibody used for immunohistochemistry is unable to distinguish between UII and URP owing to their structural homology. Hence, UII immunoreactivity represents cross‐reactivity with both UII and URP.

**Figure 3:**
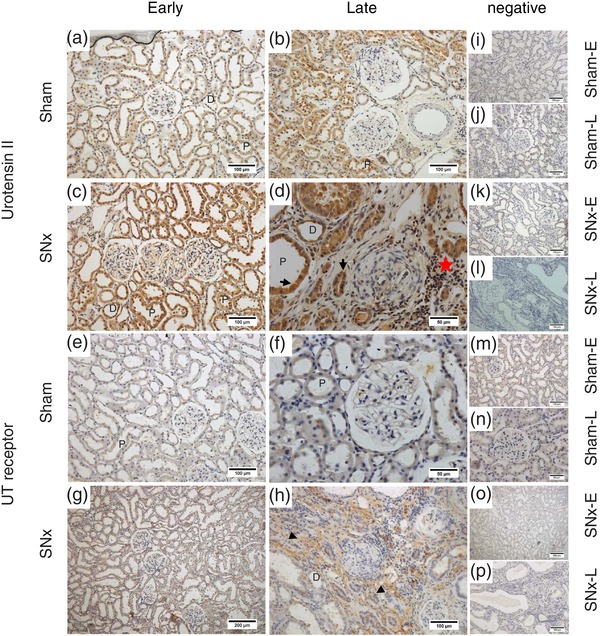
Immunolocalization of urotensin system proteins in the renal cortex as chronic kidney disease progresses. Urotensin II (UII; a–d) and urotensin receptor (UT; e–h) proteins in the cortex of sham and SNx rats during the early and late stages of renal disease. Urotensin II immunoreactivity was generally diffuse and localized to the cytoplasm of cells in the proximal tubules (P), with some staining also seen in the distal tubules (D) in the sham groups (a and b). Similar staining was seen in the early SNx group (c). In the late SNx group (d), staining was seen in the tubular epithelia (arrows), with further diffuse staining within the glomeruli and in areas of peritubular hypercellularity (red star). Diffuse UT immunoreactivity was observed in the sham groups (e and f) and in the early SNx group (g). Staining in the late SNx cohort (h) was particularly abundant in areas of peritubular hypercellularity (arrowheads). (i–p) Representative negative controls (primary antibody omitted) across each group. All images are representative and were captured at ×40 magnification with scale bars representing 50 μm (d, f, n), ×20 magnification with scale bars representing 100 μm (a–c, e, h; i–m, p) or ×10 magnification with scale bars representing 200 μm (g, o)

**Figure 4:**
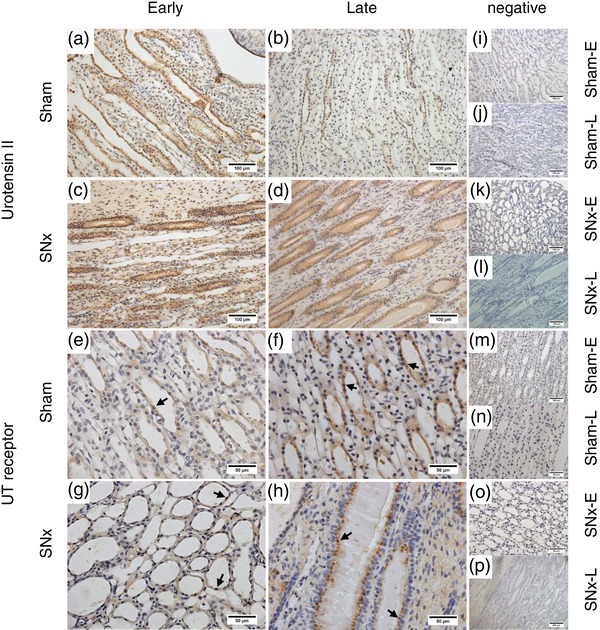
Immunolocalization of urotensin system proteins in the renal medulla as chronic kidney disease progresses. Urotensin II (a–d) and UT (e–h) proteins in the medulla of sham and SNx rats during the early and late stages of renal disease. Typically, UII immunoreactivity was localized within the cytoplasm of collecting duct epithelial cells of sham‐E (a) and sham‐L rats (b). In the SNx‐E (c) and SNx‐L (d) groups, the staining was localized in a similar manner, with additional faint staining visible in the surrounding interstitium. Expression of UT was localized within the collecting ducts (arrows). Expression of UT in SNx‐E (g) was comparable with that in sham‐E animals (e). In the SNx‐L group (h), the staining was localized in a pattern similar to that of the time‐matched shams (f) but with a pronounced apical distribution, and additional faint staining was visible in the surrounding cells. (i–p) Representative negative controls (primary antibody omitted) across each group. All images are representative and were captured at ×40 magnification with scale bars representing 50 μm (e–h, n, o), ×20 magnification with scale bars representing 100 μm (a–d, i–m) or ×10 magnification with scale bar representing 200 μm (p)

In both early (Figure 3a) and late (Figure 3b) sham rats, diffuse UII‐immunoreactive staining was seen in the proximal and distal tubules; however, there was no staining of the glomeruli. Strong positive staining was also seen in the tubules of SNx‐E (Figure 3c) and SNx‐L rats (Figure 3d), with additional staining visible within the glomeruli and in areas of proliferative expansion. Within the medulla, UII‐immunoreactive staining was seen in both the outer and inner medullary collecting ducts in all groups (Figure 4a–d). There were no apparent differences in the location of UII‐positive staining between sham (Figure 4a, b) and SNx rats (Figure 4c, d).

Very little staining for UT was observed in the cortex of sham‐E (Figure 3e), sham‐L (3f) or SNx‐E (Figure 3g) rats. In contrast, UT‐positive staining was abundant in the cortex of SNx‐L rats (Figure 3h), particularly in areas of peritubular hypertrophic expansion and within some cells in the tubules. Diffuse UT‐positive staining was observed in medullary sections from sham‐E (Figure 4e), sham‐L (Figure 4f) and SNx‐E (Figure 4g) rats. In contrast, staining in the SNx‐L cohort was associated with the apical membrane (Figure 4h).

### 3.3 Subchronic UT antagonism induces a modest delay in CKD progression

**Figure 5:**
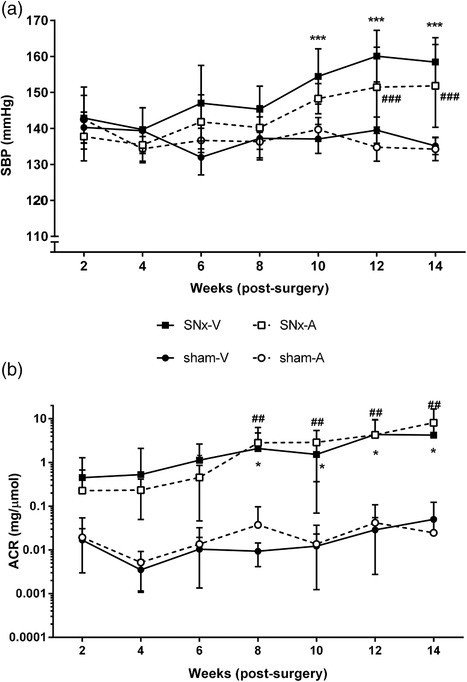
Systolic blood pressure (SBP; a) and urinary albumin:creatinine ratio (uACR; b) during subchronic treatment of SNx and sham rats with the UT antagonist SB‐611812 (30 mg kg^−1^ day^−1^) or vehicle. Data are shown as means ± SD; in panel (b), where downward error bars have negative values these have been omitted because data are plotted on a logarithmic scale. Statistical comparisons were by three‐way ANOVA with repeated measures; ^*^ *P* < 0.05, ^***^ *P* < 0.001 SNx‐V *versus* sham‐V; ^##^ *P* < 0.01, ^###^ *P* < 0.001 SNx‐A *versus* sham‐A

The SBP in the vehicle‐ and antagonist‐treated sham rats remained stable and similar throughout the 13 week period (Figure 5a). At the end of the experiment, SBP was 135.1 ± 5.9 mmHg in the sham‐V group and 134.3 ± 7.8 mmHg in the sham‐A group. In contrast, SBP increased in the SNx rats throughout the duration of the experiment (*P*_time*SNx_ < 0.001); as a result, the SNx rats had significantly higher SBP compared with the sham animals (*P*_SNx_ < 0.001). Overall, antagonist‐treated rats had significantly lower SBP than their vehicle‐treated counterparts (*P*_UT‐A_ = 0.032); this effect was attributable to the SNx‐A animals having significantly lower SBP than the SNx‐V rats (mean difference 5.9 ± 1.4 mmHg, *P*_SNx*UT‐A_ = 0.05). Closer inspection of the data revealed that SBP in the SNx‐V group diverged from that in the sham‐V rats after 10 weeks (sham‐V 137.1 ± 9.8 mmHg, *n* = 6 *versus* SNx‐V 154.4 ± 23.1 mmHg, *n* = 9, *P* < 0.001), whereas that in the SNx‐A rats did not increase compared with sham‐A rats until 12 weeks (sham‐A 134.8 ± 9.6 mmHg, *n* = 6 *versus* SNx‐V 151.5 ± 34.8 mmHg, *n* = 10, *P* < 0.001). Thus, subchronic treatment with the UT antagonist SB‐611812 delayed the increase in SBP in SNx rats by 2 weeks.

Figure 5b shows that subchronic UT antagonist treatment resulted in a comparable delay in the increase in uACR in SNx rats. Collectively, the SNx rats developed albuminuria as the study progressed. However, whereas uACR increased significantly in the vehicle‐treated SNx rats after 6 weeks [sham‐V 0.009 (0.006–0.011) mg μmol^−1^, *n* = 6 *versus* SNx‐V 0.45 (0.04–1.52) mg μmol^−1^, *n* = 11, *P* = 0.013], uACR did not increase in antagonist‐treated SNx rats until 8 weeks [sham‐A 0.009 (0.003–0.010) mg μmol^−1^, *n* = 5 *versus* SNx‐A 1.45 (0.32–4.55) mg μmol^−1^, *n* = 10, *P* = 0.006]. Hence, subchronic treatment with the UT antagonist SB‐611812 also delayed the increase in uACR in SNx rats by 2 weeks.

**Figure 6:**
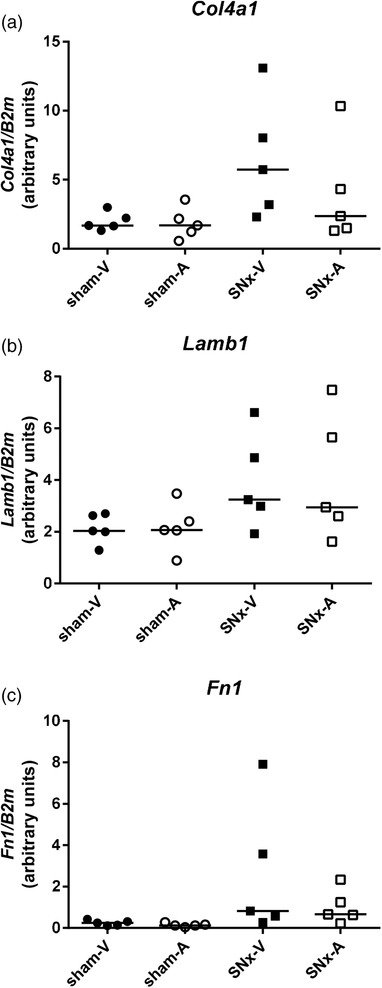
Collagen IV α1 (*Col4a1*; a), laminin‐β_1_ (*Lamb1*; b) and fibronectin 1 (*Fn1*; c) mRNA expression normalized to *B2m* in the renal cortex of SNx and sham rats after subchronic treatment with the UT antagonist SB‐611812 (30 mg kg^−1^ day^−1^) or vehicle. Compared with the SNx‐V group, 13 weeks of UT antagonist treatment in SNx rats had no effect on *Col4a1* (*P* = 0.49), *Lamb1* (*P* > 0.99) or *Fn1* (*P* = 0.83) mRNA expression. Data are shown as a scatter plot with median; statistical comparisons were by two‐way ANOVA of log_10_‐transformed data followed by Tukey's *post hoc* test

At the end of the 13 week antagonist treatment period, plasma creatinine, blood urea nitrogen and the glomerulosclerosis index were comparable between SNx‐V and SNx‐A rats and were elevated to a similar extent by comparison with their respective sham controls (Table 2). Messenger RNA expression of the glomerular basement membrane markers *Col4a1* (*P*_SNx_ = 0.013), *Lamb1* (*P*_SNx_ = 0.02) and *Fn1* (*P*_SNx_ = 0.001) (Figure 6) was elevated in SNx animals compared with the sham control animals. However, antagonist treatment did not alter the expression of these markers in SNx‐A rats compared with SNx‐V animals.

**Table 2:** Terminal plasma analysis and glomerulosclerosis index scores after subchronic treatment of subtotal nephrectomized (SNx) and sham‐operated rats with the urotensin receptor (UT) antagonist SB‐611812

	Vehicle		UT antagonist	
Parameter	Sham‐V (*n* = 5)	SNx‐V (*n* = 6)	Sham‐A (*n* = 6)	SNx‐A (*n* = 9)
Plasma creatinine (μmol l^−1^)	29.9 ± 14.3	72.6 ± 32.6	29.9 ± 18.4	92.9 ± 73.2
Blood urea nitrogen (mmol l^−1^)	2.7 ± 2.0	15.7 ± 8.6*	4.1 ± 2.0	15.6 ± 8.7*
Glomerulosclerosis index (a.u.)	0 (0–0)	1 (0–2)***	0 (0–0)	1 (0–2)***

Data are shown as means ± SD for normally distributed continuous variables or medians (interquartile range) for ordinal data. Statistical comparisons were by two‐way ANOVA with Tukey's *post hoc* tests or Kruskal–Wallis test with Mann–Whitney *post hoc* tests corrected for multiple comparisons. For clarity, comparisons are shown only between SNx and sham animals receiving the same treatment [vehicle (V) or UT antagonist (A)]. ^*^ *P* < 0.05, ^***^ *P* < 0.001 *versus* comparable sham group.

### 3.4 Expression of UT is unaffected by subchronic UT antagonism, but expression of UII is diminished in the renal cortex

**Figure 7:**
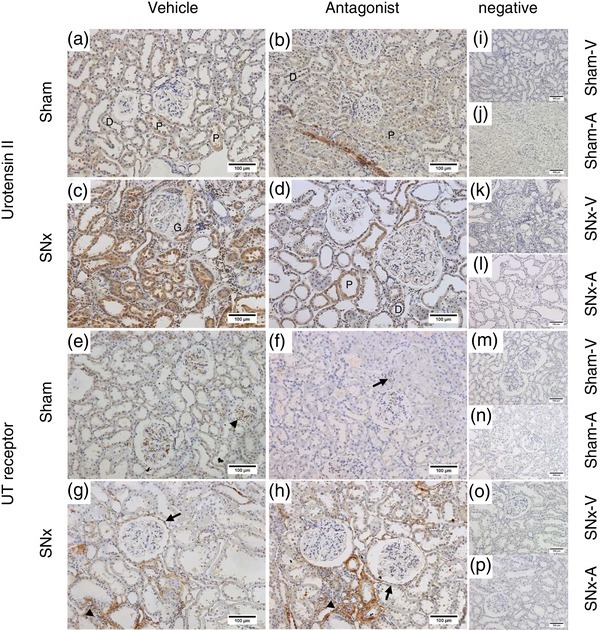
Immunolocalization of urotensin system proteins in the renal cortex of SNx and sham rats after subchronic treatment with the UT antagonist SB‐611812 (30 mg kg^−1^ day^−1^) or vehicle. Urotensin II (a–d) and UT (e–h) proteins in the cortex of sham and SNx rats receiving the UT antagonist or vehicle. Urotensin II immunoreactivity was diffuse in both sham (a, b) and SNx (c, d) groups, with the most prominent staining localized to the proximal tubules (P) (compare with distal tubules (D) and glomeruli (G)). There was limited immunostaining for UT in cortical sections from both sham groups (e, f). In SNx rats receiving vehicle (g) or UT antagonist (h), staining was observed primarily within areas of hypercellular expansion (arrowheads) rather than within intact cortical structures; some additional staining was seen in the parietal layer (arrows). (i–p) Representative negative controls (primary antibody omitted) across each group. All images are representative and were captured at ×20 magnification with scale bars representing 100 μm

**Figure 8:**
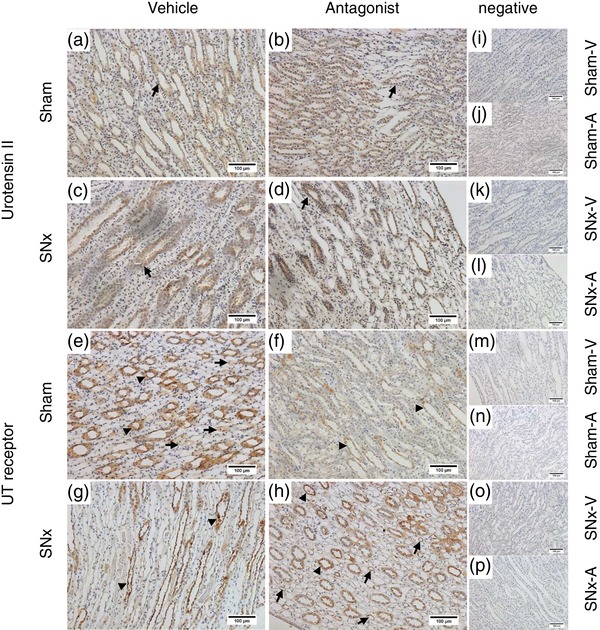
Immunolocalization of urotensin system proteins in the renal medulla of SNx and sham rats after subchronic treatment with the UT antagonist SB‐611812 (30 mg kg^−1^ day^−1^) or vehicle. Urotensin II (a–d) and UT (e–h) proteins in the medulla of sham and SNx rats receiving the UT antagonist or vehicle. In the medulla of sham rats receiving vehicle (sham‐V; a) or UT antagonist (sham‐A; b), immunohistochemical staining for urotensin II was localized primarily within the collecting duct epithelium (arrows, panels a–d). This pattern was also seen in SNx rats receiving vehicle (SNx‐V; c) or UT antagonist (SNx‐A; d). Expression of the UT receptor was consistent across sham‐V (e), sham‐A (f), SNx‐V (g) and SNx‐A (h) groups. Staining was most obvious in the collecting ducts (arrowheads, all panels); however, staining was also seen in the thin limbs of the loop of Henle (arrows in panels e and h). (i–p) Representative negative controls (primary antibody omitted) across each group. All images are representative and were captured at ×20 magnification with scale bars representing 100 μm

Urotensin II‐immunoreactive staining was mainly associated with the proximal tubules in the renal cortex of both sham‐V (Figure 7a) and sham‐A rats (Figure 7b). A similar staining pattern was evident in sections from SNx rats receiving vehicle (Figure 7c) or the UT antagonist (Figure 7d). In the medulla, UII‐immunoreactive staining was most intense within collecting duct cells across all groups (Figure 8).

In contrast to UII, there was little evidence of staining for the UT receptor in the cortex of either sham‐V (Figure 7e) or sham‐A (Figure 7f) rats. Likewise, there was little UT staining seen in normal structures of the cortex of SNx‐V (Figure 7g) or SNx‐A (Figure 7h) rats. However, UT staining was associated with areas of peritubular expansion and occasionally in the parietal layer of the Bowman's capsule in both groups of SNx rats. Staining for UT was more prominent in the medulla (Figure 8e–h), particularly within the collecting tubules and collecting ducts. UT staining was also seen in some thin limb cells, particularly in the SNx rats (Figure 8g, h).

## 4 DISCUSSION

This study has shown that expression of urotensin system proteins increases in the kidney as CKD progresses towards end‐stage renal failure, extending a previous report in which UII, URP and UT mRNA levels were quantified in the early stages of renal dysfunction in the SNx model (Mori et al., 2009). Furthermore, subchronic treatment with a UT antagonist for 13 weeks delayed the progression of CKD, albeit for a modest 2 week period. Collectively, these data suggest that upregulation of the urotensin system contributes to the progressive decline in renal function in CKD.

Immunohistochemistry revealed parallel increases in UII immunoreactivity and UT expression in the kidneys of SNx rats as renal function declined. These observations confirm a report in SNx rats at 8 weeks postsurgery (Mori et al., 2009) and now show that urotensin system expression continues to increase as renal function declines. The pattern of UII‐immunoreactive staining in the cortex and medulla of SNx rats was broadly similar to that of time‐matched sham‐operated rats and our previous observations in both ‘normal’ (Song et al., 2006) and hypertensive rats (Abdel‐Razik, Balment, & Ashton, 2008a). However, as disease progressed UII‐positive staining became apparent both in the glomeruli and in areas undergoing proliferative expansion. In addition, there was profound upregulation in the expression of UT in the cortex of SNx rats. In control conditions, we have observed only occasional staining for UT in the cortex, specifically in the glomerular arterioles and macula densa; the majority of UT staining is found in the medulla (Song et al., 2006). However, in the late SNx animals abundant UT‐positive staining was observed throughout the cortex.

The UT expression pattern in the medulla also appeared to change as SNx rats entered the later stages of renal disease. Compared with diffuse staining for UT in the cytoplasm of the collecting ducts in early SNx and sham rats, there was a pronounced apical distribution of UT in the late SNx animals. We did not measure urinary electrolyte excretion in these animals; however, UII is known to alter renal ion transport (Abdel‐Razik et al., 2008b; Song et al., 2006), and this translocation of UT receptors may therefore reflect a change in the regulation of electrolyte excretion in the later stages of renal disease.

Increased UII and UT expression have been reported in the kidneys of streptozotocin‐induced diabetic rats, in association with the accumulation of extracellular matrix components, leading to the conclusion that UII might play a causal role in renal injury and fibrosis in diabetes (Tian et al., 2008). The present study shows that increased UII and UT expression are also associated with renal dysfunction and the accumulation of extracellular matrix components in non‐diabetic CKD.

It is worth noting that although protein expression for urotensin system components was high, particularly in the kidneys from the SNx‐L group that were killed immediately before renal failure, attempts to quantify *Uts2*, *Uts2b* and *Uts2r* (UT receptor) mRNA were unsuccessful. Relative to the housekeeping gene *Ywhaz*, expression levels of *Uts2*, *Uts2b* and *Uts2r* were very low. Others have experienced similar difficulties in detecting mRNA for the urotensin system genes in rat kidneys. Sugo et al. (2003) were not able to detect *Uts2* or *Uts2b* transcripts in rat kidney using qPCR, which is consistent with the earlier work of Coulouarn et al. (1999); interestingly, both groups detected transcripts in human kidney (Coulouarn et al., 1998; Sugo et al., 2003). Tal et al. (1995) describe an absence of mRNA for *Uts2r* by RT‐PCR and Northern blot. Mori et al. (2009) reported successful amplification of the urotensin system genes; however, detection of *Uts2*, in particular, required a very high number of cycles (55 cycles), which indicates that this was not a typical assay range and is consistent with the amplification of a low number of gene copies. There is a clear mismatch between mRNA and protein levels for UII, URP and UT in the rat kidney, suggesting that changes in protein expression occur at the post‐transcriptional level.

The inverse relationship between urotensin system expression and renal function in SNx rats led us to hypothesize that UII might contribute to the progression of CKD. In order to test this hypothesis, we conducted a second experiment, in which SNx rats were treated with a UT antagonist, SB‐611812, for 13 weeks. Treatment began 1 week after the SNx surgery was complete. In these conditions, SB‐611812 treatment resulted in a 2 week delay in the increase in uACR and SBP that typifies the SNx model. Antagonist treatment did not prevent disease progression; at the end of the 13 week period, uACR, SBP, glomerulosclerosis index scores and histopathology were comparable between the vehicle‐ and antagonist‐treated SNx rats. Nonetheless, there was a delay in the onset of albuminuria and hypertension, which suggests that the urotensin system contributes to the decline in renal function in CKD. The mechanism through which upregulation of the urotensin II system results in albuminuria and hypertension is likely to involve both direct and indirect effects. Urotensin II is a potent vasoconstrictor (Ashton, 2006) and has been shown to increase fibrosis and the accumulation of extracellular matrix components in the kidney (Tian et al., 2008). Furthermore, urotensin II acts synergistically with the renin–angiotensin system, itself a major contributor to the progression of CKD (Takahashi et al., 2009), to stimulate fibrosis (Song et al., 2012) and contraction of the thoracic aorta (Wang et al., 2007). Hence, administration of SB‐611812 could have slowed disease progression by inhibiting the direct vasoactive and profibrotic actions of urotensin II or it could have blocked the synergistic action of urotensin II on angiotensin II. Further experiments are required to identify the mechanistic pathways involved.

The reason(s) for the transient nature of the effect of SB‐611812 on SBP and uACR are unclear. The preliminary pharmacokinetic profile after oral dosing at 5 mg kg^−1^ showed good bioavailability and an acceptable clearance profile. The drug dose used in the subsequent experiment, 30 mg kg^−1^ day^−1^, has been shown previously to be effective in preventing lesion of the carotid artery intima in rats after balloon angioplasty (Rakowski et al., 2005). The drug was given by gavage in both studies, although the treatment period was longer, at 28 days, in the earlier report by Rakowski et al. (2005). SB‐611812 is reported to be a competitive antagonist, with a negative log concentration of antagonist required to produce an agonist dose ratio of 2 (pA_2_) of 6.60 and 6.59 in HEK293 cells transfected with rUT and in isolated rat aorta, respectively. Given that both UII immunoreactivity and UT expression increased in the kidney as CKD progressed, it is possible that the antagonist was outcompeted by increasing local concentrations of UII or URP in the kidney. Alternatively, the presence of other factors known to contribute to disease progression in CKD might have been sufficient to stimulate continued deterioration in renal function, despite the blockade of UT receptors.

Overall, the present study has shown that the urotensin system is upregulated in non‐diabetic CKD and that expression increases as renal function declines. In particular, there is a marked increase in both UII immunoreactivity and UT expression in the renal cortex in the later stages of renal disease. These observations are consistent with stimulatory effects of UII on the deposition of extracellular matrix components and fibrosis. Subchronic treatment with a UT antagonist resulted in a modest but significant delay in disease progression in SNx rats; however, it did not prevent glomerulosclerosis. Taken together, these data suggest that the urotensin system contributes to the progression of chronic kidney disease in the rat.
